# Metabolomic Screening of Tumor Tissue and Serum in Glioma Patients Reveals Diagnostic and Prognostic Information

**DOI:** 10.3390/metabo5030502

**Published:** 2015-09-15

**Authors:** Lina Mörén, A. Tommy Bergenheim, Soma Ghasimi, Thomas Brännström, Mikael Johansson, Henrik Antti

**Affiliations:** 1 Department of Chemistry, Umeå University, SE 901 87 Umeå, Sweden; E-Mail: lina.moren@umu.se; 2 Department of Clinical Neuroscience, Neurosurgery, Umeå University, SE 901 85 Umeå, Sweden; E-Mail: tommy.bergenheim@umu.se; 3 Department of Radiation Sciences, Oncology, Umeå University, SE 901 85 Umeå, Sweden; E-Mails: soma.ghasimi@umu.se (S.G.); mikael.b.johansson@umu.se (M.J.); 4 Department of Medical Biosciences, Pathology, Umeå University, SE 901 87 Umeå, Sweden; E-Mail: thomas.brannstom@medbio.umu.se

**Keywords:** glioma, diagnosis, prognosis, blood, tumor, metabolomics, chemometrics, latent biomarkers

## Abstract

Glioma grading and classification, today based on histological features, is not always easy to interpret and diagnosis partly relies on the personal experience of the neuropathologists. The most important feature of the classification is the aimed correlation between tumor grade and prognosis. However, in the clinical reality, large variations exist in the survival of patients concerning both glioblastomas and low-grade gliomas. Thus, there is a need for biomarkers for a more reliable classification of glioma tumors as well as for prognosis. We analyzed relative metabolite concentrations in serum samples from 96 fasting glioma patients and 81 corresponding tumor samples with different diagnosis (glioblastoma, oligodendroglioma) and grade (World Health Organization (WHO) grade II, III and IV) using gas chromatography-time of flight mass spectrometry (GC-TOFMS). The acquired data was analyzed and evaluated by pattern recognition based on chemometric bioinformatics tools. We detected feature patterns in the metabolomics data in both tumor and serum that distinguished glioblastomas from oligodendrogliomas (p^tumor^ = 2.46 × 10^−8^, p^serum^ = 1.3 × 10^−5^) and oligodendroglioma grade II from oligodendroglioma grade III (p^tumor^ = 0.01, p^serum^ = 0.0008). Interestingly, we also found patterns in both tumor and serum with individual metabolite features that were both elevated and decreased in patients that lived long after being diagnosed with glioblastoma compared to those who died shortly after diagnosis (p^tumor^ = 0.006, p^serum^ = 0.004; AUROCC^tumor^ = 0.846 (0.647–1.000), AUROCC^serum^ = 0.958 (0.870–1.000)). Metabolic patterns could also distinguish long and short survival in patients diagnosed with oligodendroglioma (p^tumor^ = 0.01, p^serum^ = 0.001; AUROCC^tumor^ = 1 (1.000–1.000), AUROCC^serum^ = 1 (1.000–1.000)). In summary, we found different metabolic feature patterns in tumor tissue and serum for glioma diagnosis, grade and survival, which indicates that, following further verification, metabolomic profiling of glioma tissue as well as serum may be a valuable tool in the search for latent biomarkers for future characterization of malignant glioma.

## 1. Introduction

The World Health Organization (WHO) classification of brain tumors of neuroepithelial origin is based on histological features [1,2]. Although the classification system has been developed and improved over the years, it is still linked to some problems with possible clinical implications [3]. There is a large variation in the survival of patients with both glioblastomas (GBM) and low-grade gliomas [4,5]. The prognosis for GBM and low-grade gliomas still depends heavily on clinical factors such as age and performance status [5,6,7,8,9]. There is a great need to further improve the sub classification of malignant brain tumors and molecular pathology holds great promise. Genetic changes such as EGFR and p53 mutations have been of diagnostic importance but failed to give reliable prognostic or predictive information [10]. In anaplastic oligodendroglioma, 1p/19q deletions are associated with better response to chemotherapy [9,11] while loss of heterozygosity (LOH) 10q is shown to be a negative factor [9]. In GBM methylation of the methyl-guanin-methyl transferase (MGMT) promotor has been shown to be a positive predictive factor for temozolomide treatment in GBM [12]. More recently isocitrate dehydrogenase 1 and 2 (IDH1, IDH2) mutations have been associated with a favorable outcome for low grade gliomas in particular but also in GBM [10,13]. Therefore, discussions are ongoing for the possibility of including these markers in the WHO classification system [14]. Profiling of mutations, gene and protein expressions have recently contributed significantly to the understanding of glioma biology [15]. Downstream of the genome and proteome a plethora of low molecular weight metabolites constitute the metabolome. The metabolites and their reactions may be considered to be the functional fingerprint of protein function, genetic variation and environmental effects. So far, there are a few metabolomic studies indicating that specific metabolites detected by magnetic resonance spectroscopy (MRS) may be of prognostic value in GBM [16]. In cerebrospinal fluid (CSF), metabolomic analyses have demonstrated differences in various metabolites in different glial tumors [17]. However, reports on mass spectrometry based metabolomics studies of brain tumor tissue are sparse [18] and only a few demonstrate the diagnostic potential of metabolomics [19]. Furthermore, combined metabolomic profiling of tumor tissue and serum in the same patients are up to now unexplored. In this paper, we applied a predictive metabolomics strategy [20] to a series of consecutive primary neuroepithelial brain tumors and corresponding serum samples. Gas chromatography coupled to time of flight mass spectrometry (GC-TOFMS) and pattern recognition by means of multivariate data analysis was applied for the identification of potentially discriminative or prognostic metabolic patterns. Based on this, we show that the metabolic profiles in tumor and serum discriminate different glioma subgroups and may harbor prognostic information that may potentially play a future role as latent biomarkers in a clinical setting.

## 2. Results

### 2.1. Data Processing and Curation

From the GC-TOFMS acquired data from tumor tissue sample extracts 197 features were detected and resolved using hierarchical multivariate curve resolution (HMCR). Of those, 63 were assigned with a putative molecular identity by standard library comparisons. From the GC-TOFMS acquired data from serum sample extracts 230 features were detected and resolved. Of those, 87 could be assigned with a putative molecular identity by standard library comparisons. Unidentified features were kept in the analysis. Two features from each sample type data were found to be artifacts and thus excluded from the data. One tissue sample displayed a distinctly deviating profile compared to the others, due to an analytical error, and was excluded from further analysis resulting in a total of 80 tissue samples. Six serum samples were not properly derivatized causing erroneous data and were thus excluded from further analysis, resulting in a total of 90 serum samples included in the final analysis.

### 2.2. GBM and Oligodendroglioma Show Different Metabolic Patterns

**Figure 1:**
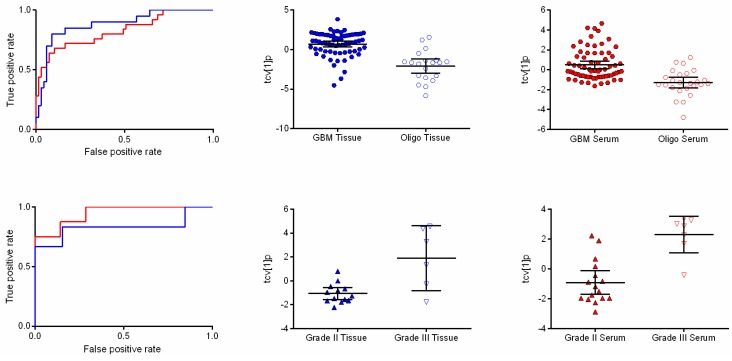
Receiver operating characteristic (ROC) curves and scatter plots of orthogonal partial least squares-discriminant analysis (OPLS-DA) scores following a seven-fold cross-validation procedure showing differences associated with diagnosis and tumor grade. (**Upper panel**) ROC curves based on the cross-validated score values from the final OPLS-DA model for the discrimination of glioblastoma and oligodendroglioma in tissue (blue line) and serum (red line) with area under the ROC curve (AUROCC) values of 0.881 (0.791–0.970) and 0.826 (0.722–0.929), respectively **(left)**. The scatter plots show the class differences between glioblastoma and oligodendroglioma based on cross-validated predictive OPLS-DA scores (tcv[1]p) for tissue (**center**) and serum (**right**). (**Lower panel**) ROC curves based on the cross-validated score values from the final OPLS-DA model discriminating between World Health Organization (WHO) grade II and III in oligodendroglioma in tissue (blue line) and serum (red line) with AUROCC values of 0.833 (0.557–1.000) and 0.946 (0.858–1.000), respectively **(left)**. The scatter plots show the class differences between grade II and grade III based on cross-validated predictive OPLS-DA scores (tcv[1]p) for tissue (**center**) and serum (**right**).

Comparing metabolic profiles from tissue between GBM and oligodendrogliomas revealed 12 significantly differentiating features (w*_average_ ± 2 standard deviations (SD)). In serum, 13 metabolic features were significantly differentially expressed. Investigation of the significance of the detected metabolic patterns by means of orthogonal partial least squares-discriminant analysis (OPLS-DA) showed that it was possible to distinguish between glioblastomas and oligodendrogliomas in both tumor and serum (A = 1 + 0 + 0, R2X = 0.39, R2Y = 0.379, Q2 = 0.341, *p* = 2.46 × 10^−8^ and A = 1 + 0 + 0, R2X = 0.25 R2Y = 0.251 Q2 = 0.223, *p* = 1.3 × 10^−5^) (Figure 1). Detected features with a suggested identity from spectral library comparison (fragmentation pattern and retention index) responsible for discriminating between the diagnoses in tumor and serum are listed in Table 1. In summary, higher levels of mannitol and phenylalanine where found in GBMs compared to oligodendrogliomas in tissue while 2-hydroxyglutaric acid, 4-Aminobutyric acid (GABA), creatinine, glycerol-2-phosphate, glycerol-3-phosphate, ribitol and myo-inositol showed higher levels in oligodendrogliomas as compared to GBM. In serum, cysteine was found at higher levels in GBMs, while lysine and 2-oxoisocaproic acid showed higher levels in oligodendrogliomas.

**Table 1:** Metabolic features altered in multivariate comparisons.

	Tissue					Serum				
Metabolite Id	RI	Corr. Diagnosis GBM *vs*. Oligo	Corr. Grade Oligo	Corr. Survival GBM	Corr. Survival Oligo	RI	Corr. Diagnosis GBM *vs*. Oligo	Corr. Grade Oligo	Corr. Survival GBM	Corr. Survival Oligo
1-Monohexadecanoylglycerol						2679		↓ *		
2-Hydroxyglutaric acid	1570.5	↓ *								
2-Oxoisocaproic acid						-	↓			
4-Aminobutyric acid (GABA)	1525.3	↓ *								
Alanine						1472.4		↑		
Aminomalonic acid	1465.0				↓ *					
Creatinine	1548.3	↓ *								
Cystine						2385.4	↑ *			
Fructose	1858.8	↓ *		↑ *						
Glycerol-2-phosphate	1714.6	↓ *								
Glycerol-3-phosphate	-	↓ *		↑ *		-				
Glycine	1305.5				↓ *					
Hexadecenoic acid						2123.6				↑
Lauric acid						1749.9	↓			
Lysine						2020.7		↓		
Maltose						2824.1		↑		↓
Mannitol	1917.5	↑ *	↑*			2029.0		↑*		
Myo-Inositol	-	↓ *		↑ *	↑	-				↑ *
Oxalic acid	1118.3		↓*							
Phenylalanine	1621.0	↑ *				1722.0				
Ribitol	1708.2	↓ *		↑ *	↑ *					
Serine	1358.4		↑							
Spermidine	2244.7				↑ *					
Sterol	2864.5				↓					
Threonic acid	1551.6		↑							
Threonic acid-1,4-lactone						1472.2		↑		

The Metabolite id column show putative identities of all resolved features altered in the multivariate models based on spectral library comparison (fragmentation pattern and retention index). RI denotes retention index. The Corr. Diagnosis column shows the features affected by diagnosis (GBM *vs.* oligodendroglioma) where the arrows denote if the metabolic feature is elevated (↑) or lowered (↓) in GBM compared to oligodendrogliomas. The Corr. Grade column shows the feature affected by different grades (II and III) in oligodendrogliomas, the arrows illustrate if the metabolic feature is elevated (↑) or lowered (↓) in grade III compared to grade II. The column Corr. Survival GBM, show the features that differ between long and short survival in glioblastoma and the Corr. Survival Oligo column show the metabolic features that differ in relation to survival time in oligodendrogliomas. The arrows illustrate if the metabolic feature is elevated (↑) or lowered (↓) in long survival patients as compared to short survival patients. * denote a significant *p*-value (<0.05) calculated using Mann–Whitney U test.

### 2.3. Metabolic Differences between Oligodendroglioma WHO Grade II and III

For the comparison between oligodendroglioma grade II and grade III, 10 resolved features fulfilled the significance criteria in tissue and provided a separation between the two sample classes in an OPLS-DA model (A = 1 + 0 + 0, R2X = 0.579, R2Y = 0.505, Q2 = 0.434, *p* = 0.01) (Figure 1). Serine, threonic acid and mannitol were all elevated in in oligodendroglioma grade III, while oxalic acid was elevated in oligodendroglioma grade II. In serum, 12 resolved features as a pattern in an OPLS-DA model provided a significant difference between oligodendroglioma grade II and grade III (A = 1 + 0 + 0, R2X = 0.405, R2Y = 0.589, Q2 = 0.589, *p* = 0.0008) (Figure 1). Mannitol, maltose, threonic acid-1,4-lactone and alanine were all higher in relative concentration in grade III oligodendrogliomas, while lysine and 1-monohexadecanoylglycerol were found in lower relative concentrations in grade III as compared to grade II oligodendrogliomas (Table 1).

### 2.4. Metabolic Profiles Associated with Survival

**Figure 2:**
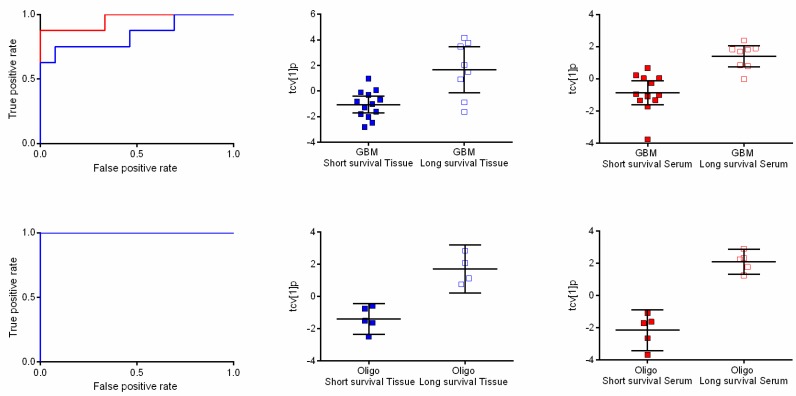
Receiver operating characteristic (ROC) curves and scatter plots of orthogonal partial least squares-discriminant analysis (OPLS-DA) scores following a seven-fold cross-validation procedure showing differences between long and short survival time. (**Upper panel**) ROC curves based on the cross-validated score values from the final OPLS-DA model for the discrimination of short survival time compared to long survival time in patients with glioblastomas in tissue (blue line) and serum (red line) with ROC values of 0.846 (0.647–1.000) and 0.958 (0.870–1.000), respectively **(left)**. The scatter plots show class differences between short survival time and long survival time based on cross-validated predictive OPLS-DA scores (tcv[1]p) for tissue (**center**) and serum (**right**). (**Lower panel**) ROC curves based on the cross-validated score values from the final OPLS-DA model for the discrimination of short survival time compared to long survival time in patients with oligodendrogliomas in tissue (blue line) and serum (red line). AUROCC values for survival in oligodendroglioma were calculated to 1 (1.000–1.000) for both tissue and serum **(left)**. The scatter plots show class differences between short survival time and long survival time based on cross-validated predictive OPLS-DA scores (tcv[1]p) for tissue (**center**) and serum (**right**).

In GBM, the metabolic profiles of tumor samples from patients surviving long after diagnosis (≥3 years) were compared to patients that died shortly after diagnosis (≤4 months). Based on seven resolved features passing the significance criteria, OPLS-DA provided a significant separation associated with time of survival (A = 1 + 0 + 0, R2X = 0.669, R2Y = 0.474, Q2 = 0.427, *p* = 0.006) (Figure 2). Interpretation of the model revealed that glycerol-3-phoshate, myo-inositol, ribitol and fructose increased in level with long survival. Furthermore, we detected a significant association with survival time in serum samples from the same patients in an OPLS-DA model based on five resolved features (A = 1 + 0 + 0, R2X = 0.536, R2Y = 0.572, Q2 = 0.478, *p* = 0.004; Figure 2). Unfortunately, it was not possible to obtain a suggested identity for any of the affected features found in serum. The same comparison was carried out in patients with oligodendroglioma. In tumor tissue, eight resolved features passed the significance criteria and provided a significant metabolic pattern (OPLS-DA model) in relation to time of survival (A = 1 + 0 + 0, R2X = 0.56, R2Y = 0.796, Q2 = 0.767, *p* = 0.01; Figure 2). High levels of ribitol, myo-inositol and spermidine were associated with long survival, while high levels of glycine, aminomalonic acid and highly likely an unidentified sterol were associated with short survival time. A complete separation with respect to survival time could also be seen in serum (Figure 2). The final OPLS-DA model was based on 13 resolved features, which together formed a significant metabolic pattern (A = 1 + 0 + 0, R2X = 0.521, R2Y = 0.909, Q2 = 0.855, *p* = 0.001). In this model, myo-inositol and hexadecenoic acid were associated with long survival time. All features with a putative identity significant in oligodendroglioma survival can be viewed in Table 1. AUCROCC analyses of the extracted metabolic patterns visualized as the OPLS-DA score values following a seven-fold cross-validation procedure for GBM in tumor and serum gave ROC values of 0.846 (0.647–1.000) and 0.958 (0.870–1.000), respectively (Figure 2). In oligodendroglioma, the corresponding ROC values reached the value 1 (1.000–1.000) in both tumor and serum.

### 2.5. Pathway Analysis

By processing the features of interest using their putative metabolite identity (Table 1) utilizing the IPA^®^ platform (Ingenuity Systems, Inc), a number of canonical pathways in serum and tissue were identified, with amino and fatty acid metabolism ending up among the top ranked pathways. Metabolites involved in theses pathways as well as other metabolites of interest described in Table 1 with regards to tumor biology were selected for further discussion.

## 3. Discussion

### 3.1. Metabolomic Differences Associated with Diagnoses and Grading

The present study is one of the first to demonstrate metabolomic pattern differences in tumor tissue between different neuroepithelial tumors as well as the potential prognostic information obtained utilizing mass spectrometric methods. In addition, it is the first study to demonstrate the metabolomic pattern differences in corresponding serum samples from the very same patients. Previously, Cueller-Baena demonstrated differences between different pediatric brain tumors by High-Resolution Proton Magnetic Angle Spinning Spectroscopy (HR-MAS) [19]. Using H-MRS, Law *et al.* demonstrated that MR spectroscopy could be of value to predict the grading of glial tumors [21]. Also using HR-MAS NMR, Erb *et al.* could distinguish different grades of oligodendrogliomas [18]. By mass spectrometric methods Chinnaiyan *et al.* have shown that the metabolomic profile in tissue differs between different grades of glioma [22], a finding supported by the paper from Nakamizo *et al.* reporting differences in CSF related to the grade of astrocytoma/GBM [17].

From a biological point of view, it could appear quite obvious that there should be a difference in the metabolism of different primary brain tumors [23]. In our study, there is a significant difference between GBM and oligodendrogliomas, especially in tissue but interestingly also in serum. In tissue, a pattern based on 12 resolved features and in serum 13 resolved features were included in the multivariate model. Whether this difference is related to the tumor grade itself or to the histological type cannot be concluded from our analyses. However, when analyzing oligodendrogliomas a distinct metabolic pattern was detected related to grade, *i.e.*, grade II and III. This finding could indicate that changes in the metabolome are correlated to not only the histology type but also to the grade of the tumors.

### 3.2. The Metabolome as Prognostic Factor

Grading of the tumors should according to the aims of the WHO classification be related to the prognosis [2]. However, for many histopathological entities there are large variations in survival. For example, in GBM the survival can vary between 0.4 to 142 months in larger series [4] and for low-grade gliomas from 0.2 to 16 years [5]. In a clinical setting, this is unsatisfactory. Quon *et al.* have by repeated MRS in glioma patients undergoing surgery and radiotherapy demonstrated that changes in choline could provide prognostic information [24]. In a microdialysis study, our group has previously investigated the metabolomic alterations in GBM during the early course of radiotherapy [25]. In that study, significant changes in metabolic patterns were disclosed. Majos *et al.* analyzed MRS spectra in patients with high-grade astrocytomas (grade III and GBM) and found prognostic information [26]. Although they did not identify any specific metabolites, their study points to the possibility of utilizing a metabolic signature or pattern for prognostication. Our analysis showed that were associations between the metabolome and time of survival in tumor and serum of both GBM and oligodendrogliomas and that there was a break-point for a high prognostic value based on changes in the metabolome for patients with GBM living shorter than four months, and longer than three years. A prognosis of less than four months survival might indicate that an aggressive treatment should be avoided, while a prognosis better than three years may support a more active approach. For oligodendrogliomas, the corresponding break-points were two and three years, respectively. If further verifications of these findings are successful in separate sample cohorts, this methodology might possess a potential means to extract latent variables holding prognostic information regarding glioma survival.

### 3.3. Metabolic Pathways and Specific Metabolites of Interest

In general, we have found it hard to corroborate our findings to the existing literature due to the lack of metabolomics data regarding gliomas. Although there are an increasing number of publications based on MRS and NMR reporting on single or a few metabolites, there are few studies on metabolomics utilizing mass spectrometric methods in brain tumors. By utilizing the IPA^®^ pathway analysis platform, we could identify a number of potential pathways, including amino and fatty acid metabolism, or metabolic entities that the detected metabolites of interest are involved in. Since the high grade of complexity in tumor metabolism possesses a major challenge when it comes to mechanistic interpretations, we found it difficult to draw any major mechanistic conclusions based on the pathway analysis in this limited material Nevertheless, some of the putatively identified metabolites found in our study can be interpreted in a biological context and are discussed below.

Mannitol was found at higher levels in GBM compared to oligodendroglioma and in oligodendroglioma grade III compared to oligodendroglioma grade II. In clinical practice mannitol is used temporarily during surgery to reduce brain edema. Almost all patients, except for a few with low-grade tumors, received 200–300 mL mannitol approximately 1–2 h before resection of the tumor. Mannitol is a large molecule that normally does not pass the blood–brain barrier, therefore, it is not surprising that we found higher levels of mannitol in the high-grade tumors where a more or less defect blood–brain barrier is to be expected. Peeling *et al.* have previously demonstrated that mannitol was found in glioma tissue only when the tumors analyzed showed a contrast enhancement on MR indicating a disruption of the blood-brain barrier [27].

Creatinine was also found as significantly altered between GBM and oligodendroglioma. It is a breakdown product of creatine phosphate, which can be used as a reserve of high-energy phosphates for the brain, anaerobically phosphorylating ADP to ATP [28]. Creatine phosphate is likely to be consumed to a higher extent by the more aggressive tumors leading to lower levels of the creatine phosphate breakdown product, creatinine. This could explain why we see lower levels of creatinine in tissue from GBM tumors as compared to oligodendroglioma.

We also found that levels of GABA in tumor were lower in GBM compared to oligodendroglioma. GABA is an important inhibitory neurotransmitter. The number of publications reporting on GABA in glioma is sparse and provides conflicting information. One study by Faria *et al.* showed that GABA was detectable in low-grade astrocytomas and normal brain, however they failed to detect GABA in high grade tumors [29], while another study by Biachi *et al.* found increased levels of GABA in GBM as compared to normal brain using microdialysis [30]. Another neurotransmitter, glutamate, is a substrate for GABA and an intermediate in the glutamine conversion to oxaloacetate in the amino acid, nucleotide and lipid synthesis [31]. Glutamate takes part in the energy supply, and has an important role as an excitotoxic substance promoting glioma invasiveness [32,33]. Several studies have demonstrated, by NMS in tissue or in the extracellular space by microdialysis, that the glutamate concentration is higher in more malignant gliomas, or in gliomas compared to normal brain [34,35,36]. In this study, we did not find any significant differences in glutamate levels, however, we had no possibility to compare low-grade astrocytomas with GBM.

Glycerol-3-phosphate is the backbone of triglycerides and glycerophospholipids and is also involved in the fatty acid oxidation cycle generating NADH. Accordingly, we found that the glycerol-3-phosphate level was lower in GBM compared to oligodendrogliomas as well as lower in short surviving patients, which would be expected in highly proliferating tumors.

Myo-inositol is an interesting metabolite. Previous studies have reported that low levels of myo-inositol are associated with higher aggressiveness of the glioma phenotype [37,38]. Kinoshita *et al.* and Faria *et al.* also found that inositol was lower in GBM as compared to astrocytoma grade II and III, while Wright and fellows found that the myo-inositol level in GBM where similar to the level in astrocytoma grade III but lower than in grade II [29,36,39]. We can confirm those findings in our study where we found that the level of myo-inositol was significantly lower in GBM as compared to oligodendrogliomas and that long survival patients from both tumor types have higher levels of myo-inositol than patients that die shortly after diagnosis. Myo-inositol is an activator of protein C kinase [37]. The activation of PKC contribute to tumor cell survival and proliferation and has shown to be involved in the progression of malignant gliomas [40].

When comparing metabolic profiles in patients that lived long after diagnosis compared to patients that died shortly after diagnosis we found glycine to be of interest. Glycine has been detected by MRS at higher concentrations in high grade gliomas and therefore been suggested as a possible diagnostic marker [41]. In our study, glycine were higher in patients that died shortly after diagnosed with oligodendroglioma supporting that higher concentrations of glycine is associated with a worse prognosis. Unfortunately, no correlation of glycine with survival in GBM could be found in our material.

### 3.4. Multivariate Metabolic Patterns and Latent Biomarkers

Analyzing the metabolome is a complex and difficult task. We believe that one should keep the discussion of metabolic differences between different histopathological types of tumors apart and separate from the discussion of metabolism within a specific type of tumor. Merging all gliomas into one group only taking the grade into account may result in confusing conclusions. It is also important that the differences disclosed are based on a multivariate analysis, which does not necessarily mean that specific metabolites in the statistical model are of prime importance by themselves, but rather the combined pattern together with other defined metabolites. We consider the main findings of the study to be that there is evidence suggesting that metabolic patterns in both tumor and serum contain information that potentially can be used as diagnostic or even as prognostic latent biomarkers in gliomas. Although very interesting, we also realize the need to verify these findings in separate materials with higher sample numbers in order to get a correct measure of the predictive ability as well as a clearer picture of the clinical value of the extracted latent biomarkers.

## 4. Method

### 4.1. Samples

Snap-frozen tumor tissue from 81 gliomas was included in the study. The series was consecutively collected from patients that underwent open resection and day-time surgery during 2004 to 2008. Fifty-seven patients were diagnosed according to the WHO classification with GBM, 4 patients were diagnosed with grade II and III astrocytomas and 20 patients with oligodendroglioma of WHO grade II and III. All samples were retrospectively reviewed and classified according to WHO 2007 [1]. Tissue for diagnostic analyses was always collected first and only if the amount of tissue was enough, samples for research were collected. The collected tissue was snap-frozen in 2 mL polypropylene vials (Sarstedt AG & Co, Nümbrecht, Germany) in liquid nitrogen at the surgical theatre immediately following resection from the patient. Serum samples from the same patients were collected in 10 mL plain glass blood-tubes (BD Vacutainer^®^) spun down, fractionated and frozen in 2 mL polypropylene vials (Sarstedt AG & Co.) at −20 °C within 45 min. Serum was collected from ten additional patients diagnosed with GBM and from five more patients with oligodendrogliomas. The four patients diagnosed with astrocytoma grade II and III were excluded from further analyses due to the low number making statistical analyses unreliable. All collected samples were transferred to −80 °C within one week.

All patients included gave their informed consent to participate and the study was approved by the Ethics committee of Umeå University.

### 4.2. Sample Preparation and GC-TOFMS Analysis

One milliliter extraction solution consisting of chloroform (20%), methanol (60%) and water (20%) with 11 IS (7 ng/μL) was added to 15 mg of tissue. Tissue samples were kept frozen on dry ice until the extraction solutions were added. Two tungsten beads were placed in each sample tube and the tissue samples were milled for 2 min, 30 Hz using a MM301 vibration Mill (Retsch GmbH & Co. KG, Haan, Germany) before centrifuged for 15 min, 4 °C, 14 000 rpm. 200 μL of the collected supernatant was transferred to GC vials and evaporated to dryness. The serum samples were thawed in room temperature for 30 min before addition of 900 μL extraction solution consisting of methanol (90%) and water (10%) with 11 IS (7 ng/μL) to 100 μL serum. The serum samples were extracted using the same approach as for the tissue samples, except for the tungsten beads. The samples were then methoxymated with 30 μL of methoxyamine solution in pyridine (15 μg/μL) and left standing at room temperature for 16 h before trimethylsilylation with 30 μL of MSTFA. After 1 h, 30 μL of heptane (containing 0.5 μg of methyl stearate) was added. One microliter of derivatized sample was injected splitless by an Agilent 7683 Series autosampler (Agilent, Atlanta, GA, USA) in randomized order into an Agilent 6980 GC equipped with a 10 m × 0.18 mm i.d. fused-silica capillary column chemically bonded with 0.18 μm DB5-MS stationary phase (J&W Scientific, Folsom, CA, USA). The injector temperature was set to 270 °C. Helium was used as carrier gas at a constant flow rate of 1 mL/min through the column. The purge time was set to 60 s at a purge flow rate of 20 mL/min and an equilibration time of 1 min for every analysis. Initially, the column temperature was kept to 70 °C for 2 min and then increased to 320 °C at 30 °C/min, where it was kept for 2 min. The column effluent was introduced into the ion source of a Pegasus III TOFMS (Leco Corp., St Joseph, MI, USA). The transfer line temperature was set to 250 °C and the ion source temperature to 200 °C. Ions were generated by a 70 eV electron beam at a current of 2.0 mA. Masses were acquired from *m*/*z* 50 to 800 at a rate of 30 spectra/s, and the acceleration voltage was turned on after a solvent delay of 165 s. The stable isotope-labeled internal standard compounds (IS) [^13^C_5_]-proline, [^2^H_4_]-succinic acid, [^13^C_5_,^15^N]-glutamic acid, [1,2,3-^13^C_3_]-myristic acid, [^2^H_7_]-cholesterol and [^13^C_4_]-disodium α-ketoglutarate were purchased from Cambridge Isotope Laboratories (Andover, MA). [^13^C_12_]-sucrose, [^13^C_4_]-palmitic acid and [^2^H_4_]- butanediamine 2HCl were from Campro (Veenendaal, The Netherlands). [^13^C_6_]-glucose was from Aldrich (Steinheim, Germany) and [^2^H_6_]-salicylic acid was from Icon (Summit, NJ, USA). Stock solutions of the IS were prepared either in purified and deionized water (Milli-Q, Millipore, Billerica, MA, USA) or in methanol (J.T. Baker, Deventer, The Netherlands) at a concentration, 0.5 μg/μL. Methyl stearate was purchased from Sigma (St. Louis, USA). *N*-Methyl-N-trimethylsilyltrifluoroacetamide (MSTFA) with 1% trimethylchlorosilane (TMCS) and pyridine (silylation grade) were purchased from Pierce Chemical Co. Heptane was purchased from Fischer Scientific (Loughborough, UK).

### 4.3. Data Processing

To be able to relatively quantify and provide a putative identity for all detected individual features, the acquired GC-TOFMS data were processed by applying HMCR [42,43]. HMCR uses a multivariate approach for generating pure chromatographic signatures together with corresponding mass spectra for all detected feature peaks. In this way multiple sample comparisons based on the whole metabolic profile will be greatly facilitated. For this, NetCDF files of the raw acquired GC-TOFMS data were exported to MATLAB 7.11.0 (R2010b) (Mathworks, Natick, MA, USA) where baseline correction, alignment, time-window settings were carried out before applying HMCR time-window wise to resolve the individual feature peaks. All data processing including HMCR was done using in-house developed scripts. For the tissue data, the chromatograms were divided into 64 time windows from which 197 chromatographic peaks (features) were resolved resulting in a data matrix (**X**) were each row represents one patient and each column represents one feature, e.g. metabolite. Similarly, for the serum data, the chromatograms were divided into 67 time windows from which 230 chromatographic peaks were resolved. For each feature in each sample the area under the chromatographic peak, the relative concentration, was calculated. All peak areas were normalized using the peak areas from the 11 internal standards. The detected features’ mass spectral profile and retention indices were compared to spectra in an in-house spectral library of metabolite standards and the NIST library 2.0 (as of 31 January 2001) to provide a putative identity for each individual feature. This was followed by a manual inspection and curation of the data to further resolve co-eluting compounds and to correct for split peaks.

### 4.4. Pattern Recognition and Statistical Analysis

**Figure 3:**
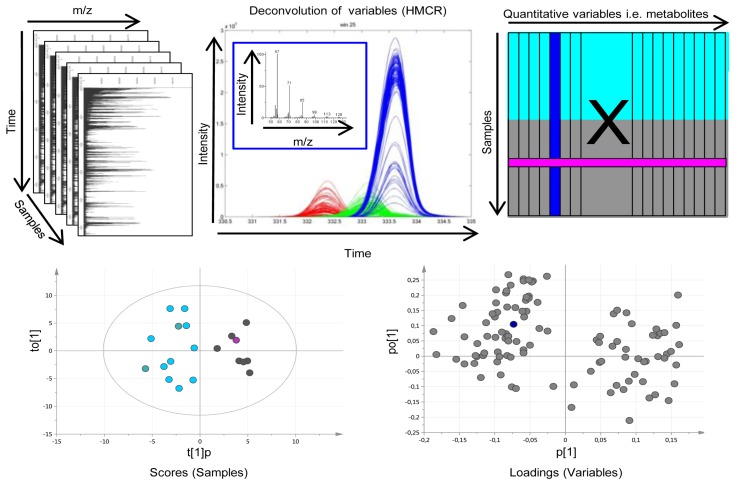
Overview of the metabolomic workflow. (**Upper panel. *Left***) Raw gas chromatography-time of flight mass spectrometry (GC-TOFMS) data for the analyzed samples makes up a three dimensional matrix with the *Time* axis being retention time or index for each metabolite linked to the elution from the chromatographic system, the mass to charge (*m/z)* axis being the mass over charge ration for the molecular fragments detected by the mass spectrometer and the *Samples* axis being the analyzed samples. (***Middle***) To obtain pure chromatographic and spectral profiles for relative quantification and identification of metabolites the raw GC-TOFMS data was processed by hierarchical multivariate curve resolution (HMCR), which is a multivariate deconvolution technique especially developed to resolve complex GC-MS based metabolomics data from multiple samples to make it suitable for multiple sample comparisons by means of e.g. pattern recognition approaches. (***Right***) The area under each resolved metabolite peak makes up the variables of the resulting data matrix (X) used as input for further pattern recognition and statistical analysis. Each column of X represents one resolved metabolite peak over all samples (rows of X). Chemometric bioinformatics based pattern recognition is applied to, X.; e.g. for investigating the difference between two sample classes (turquoise and grey in X). (**Lower panel. *Left***) The sample variation of X is projected in the model scores allowing interpretation of sample distribution patterns. Each symbol in the scores plot represents one sample described by all variables/metabolites (columns of X). As an example, the pink sample symbol relates to the pink row of X. (***Right***) The variable/metabolite variation is projected in the model loadings allowing interpretation of sample distribution patterns and explanation of variable contribution to patterns in seen in scores. Each symbol in the loading plot represents on variable/metabolite. As an example, the blue symbol relates to the blue column of X as well as the blue resolved metabolite profile in the upper middle frame.

Pattern recognition utilizes multivariate projection methods to extract and verify co-varying patterns or signatures of variables that are significant for explaining systematic variation in experimental data. In metabolomics, pattern recognition works to compress the variable space, *i.e.*, the detected and relatively quantified metabolic features, into a few latent variables, e.g., principal components, explaining the majority of the systematic variation in the data. In this way interpretation of changes in metabolic signatures as well as detection of robust and relevant sample patterns caused by those signature changes are largely facilitated. In this work, processed metabolomics data from tissue and serum samples were analyzed separately using different pattern recognition approaches. In a first step, principal component analysis (PCA) [44] was applied to get an unsupervised overview of the variation in the data and to detect deviating samples, so-called outliers. For further multivariate sample comparisons with the aim to look for differences between pre-defined sample classes, orthogonal partial least squares-discriminant analysis (OPLS-DA) [45] was used. OPLS is a supervised multivariate regression method allowing a separation of the variation into predictive variation (related to the response(s) of interest) and orthogonal variation (variation unrelated to the response(s) of interest). This has been shown to facilitate the interpretation of complex multivariate data and the interactions therein. The combined data processing and pattern recognition procedure can be overviewed in Figure 3. Initially, the diagnostic potential of the metabolic profiles in terms of discriminating between GBMs and oligodendrogliomas was evaluated. The low grade astrocytomas (grade II and III) were excluded from further evaluation due to the low number of samples making statistical analysis unfeasible. Then differences in tumor grade were investigated comparing samples from oligodendroglioma patients with grade II and grade III tumors, respectively. Finally, metabolic patterns associated with survival time were investigated in glioblastomas and oligodendrogliomas separately. From the calculated OPLS-DA models, model weight values (w*), *i.e.*, variable contribution values for the pre-defined sample class separations, were extracted and only variables related to the class separation were included in the final OPLS-DA models (w*_average_ ± 2 SD). Furthermore, a Mann–Whitney U-test was used to calculate a probability value (*p*-value) for each included metabolite in relation to the class separations of interest. All models were validated using cross-validation and p-values for the cross-validated model were calculated using CV-ANOVA [46]. A seven-fold cross-validation procedure was applied using 1/7 of the data as the test set, while the remaining 6/7 of the data were modeled and then repeating this seven times. Furthermore, for all OPLS-DA models the number of latent variables (OPLS components) (A), the variation described in the metabolite data (R2X), the between class variation described (R2Y) and the between class variation predicted based on cross-validation (Q2) were reported. When comparing time of survival in GBM, patients that died shortly after diagnosis (≤4 months) were compared to patients that lived long after diagnosis (≥3 years). For oligodendrogliomas, patients that died within 2 years after diagnosis (short survival) were compared to patients that lived for more than 3 years (long survival). Survival groups were selected based on retrospective data from our institution with an expected 4 months survival in GBM of 65% and 3 year survival of only 8%. In oligodendroglioma grade II, 2- and 3-year survival is expected to be 78% and 75%, respectively. For patients with oligodendroglioma grade III expected survival of 2 and 3 years is 50% and 50%, respectively. However, in our consecutively collected material, we did not have enough patients within any clinically relevant time limits. Instead, we had two almost equally sized groups of patients; one group that died within two years of diagnosis and the other group that lived longer than 3 years, which is why those limits were the most inherent to get reliable statistics. All pattern recognition analysis, including cross-validation and CV-ANOVA, was performed in SIMCA (version SIMCA-P + 13.0; Umetrics, Umeå, Sweden). Model plots were created using SIMCA or GraphPad Prism (5.04; GraphPad Software Inc., La Jolla, CA, USA) in combination with Adobe Illustrator CS5 (15.0.0; Adobe Systems Inc., San Jose, CA, USA). To summarize the results, Receiver Operating Characteristic (ROC) curves were calculated for the detected significant metabolic patterns associated with survival. This way of utilizing ROC curves for metabolic patterns as compared to the conventional way using single markers is novel and makes it possible to evaluate the diagnostic and prognostic potential of metabolic patterns or signatures in a way that is familiar to the clinical community. The ROC calculations were performed in ROCCET: ROC Curve Explorer & Tester (www.roccet.ca) [47].

### 4.5. Pathway Analysis

Resolved features with a putative identity surviving the criteria for significance described previously were subjected to pathway analysis, using IPA^®^ (Ingenuity Systems, Inc, Reawood City, CA, USA). Top canonical pathways and biological functions were investigated. Detected metabolites in pathways relevant for tumor biology were selected for further evaluation and discussion.
